# Effects of Doxycycline on gene expression in *Wolbachia *and *Brugia malayi *adult female worms in vivo

**DOI:** 10.1186/1423-0127-19-21

**Published:** 2012-02-09

**Authors:** Ramakrishna U Rao, Yuefang Huang, Sahar Abubucker, Michael Heinz, Seth D Crosby, Makedonka Mitreva, Gary J Weil

**Affiliations:** 1 Infectious Diseases Division, Department of Internal Medicine, St. Louis, Missouri, USA; 2 The Genome Institute, Washington University School of Medicine, St. Louis, Missouri, USA

**Keywords:** Doxycycline, *Brugia malayi*, *Wolbachia*, Filariasis, Gene expression, Microarray

## Abstract

**Background:**

Most filarial nematodes contain *Wolbachia *symbionts. The purpose of this study was to examine the effects of doxycycline on gene expression in *Wolbachia *and adult female *Brugia malayi*.

**Methods:**

*Brugia malayi *infected gerbils were treated with doxycycline for 6-weeks. This treatment largely cleared *Wolbachia *and arrested worm reproduction. RNA recovered from treated and control female worms was labeled by random priming and hybridized to the Version 2- filarial microarray to obtain expression profiles.

**Results and discussion:**

Results showed significant changes in expression for 200 *Wolbachia *(29% of *Wolbachia *genes with expression signals in untreated worms) and 546 *B. malayi *array elements after treatment. These elements correspond to known genes and also to novel genes with unknown biological functions. Most differentially expressed *Wolbachia *genes were down-regulated after treatment (98.5%). In contrast, doxycycline had a mixed effect on *B. malayi *gene expression with many more genes being significantly up-regulated after treatment (85% of differentially expressed genes). Genes and processes involved in reproduction (gender-regulated genes, collagen, amino acid metabolism, ribosomal processes, and cytoskeleton) were down-regulated after doxycycline while up-regulated genes and pathways suggest adaptations for survival in response to stress (energy metabolism, electron transport, anti-oxidants, nutrient transport, bacterial signaling pathways, and immune evasion).

**Conclusions:**

Doxycycline reduced *Wolbachia *and significantly decreased bacterial gene expression. *Wolbachia *ribosomes are believed to be the primary biological target for doxycycline in filarial worms. *B. malayi *genes essential for reproduction, growth and development were also down-regulated; these changes are consistent with doxycycline effects on embryo development and reproduction. On the other hand, many *B. malayi *genes involved in energy production, electron-transport, metabolism, anti-oxidants, and others with unknown functions had increased expression signals after doxycycline treatment. These results suggest that female worms are able to compensate in part for the loss of *Wolbachia *so that they can survive, albeit without reproductive capacity. This study of doxycycline induced changes in gene expression has provided new clues regarding the symbiotic relationship between *Wolbachia *and *B. malayi*.

## Background

Like many filarial nematodes, *Brugia malayi *contain intracellular *Wolbachia *bacteria (*w*Bm) [1-3]. These obligatory endosymbionts are essential for larval growth and development and for adult worm reproduction and survival [4-7]. They multiply by binary fission and are vertically transferred to successive generations of worms. Metabolic interdependence of endosymbionts and their hosts is common [8-10]. Two fundamental questions remain in this mutualistic relationship: what do *w*Bm contribute to the nematodes and what do the worms provide for the bacteria?

*w*Bm have been considered to be important biological targets for drug development against filarial nematodes [11,12]. Many antibiotics have been tested *in vitro *and *in vivo *against *w*Bm and filarial worms. Of these, tetracycline and related drugs have been shown to be effective *in vitro*, in animal models, and in humans against filarial infections [6,13-18]. Tetracyclines also have prophylactic activity, and they can cause a male-biased sex-ratio [19-21]. Doxycycline (Doxy) reduces microfilaremia and adult worm burdens by sterilizing adult worms and eventually killing them (in filarial species containing *Wolbachia*), either alone or in conjunction with diethylcarbamazine (DEC) or ivermectin [22-26]. In many of these studies, antibacterial effects of the drugs precede the effects on worms. In contrast, microfilariae and adult worms of filarial species that lack *Wolbachia *such as *Acanthocheilonema viteae *and *Loa loa *are not affected by tetracyclines [27,28]. Therefore the effect of tetracyclines in filarial worms with *w*Bm is believed to be related to their effect on the bacteria and not due to direct effects on the worms. Some authors have suggested that *w*Bm may provide key molecules or metabolic functions that are essential for survival and reproduction in species that are *w*Bm dependent [29,30]. Doxycycline primarily affects bacterial growth and survival by inhibiting the binding of amino-acyl tRNA in ribosomes which blocks protein synthesis [31,32]. At higher concentrations, doxy has been shown to affect mitochondrial function [33,34].

Despite their occurrence in several filarial nematodes and their emergence as a therapeutic target, little is known regarding biological interactions between *w*Bm and filarial worms [29,30]. A differential display PCR-based approach has been used to look at changes in gene expression in *Litomosoides sigmodontis *after tetracycline treatment [35]. A number of genes showed increased expression after doxy including a phosphate permease gene (Ls-ppe-1) which may be essential for embryo development. The genomes of *w*Bm and Bm provided more clues regarding potential mechanisms of interdependence [29,36,37]. For instance, comparative genome studies suggested that *w*Bm contains pathways for production of riboflavin, flavin adenine dinucleotide, heme, and nucleotides that are lacking in *B. malayi*. In addition, since the *w*Bm genome lacks complete biochemical pathways for de novo synthesis of biotin, coenzyme A, NAD, ubiquinone and folate, filarial worms may provide these and other molecules that are required for bacterial growth. *w*Bm also lack synthetic pathways for most amino acids [29], and they probably obtain amino acids from their filarial hosts [36].

In this study we have examined the effects of 6-week doxy treatment on *w*Bm and Bm, and used V2 Bm microarray to observe changes in expression profiles of many genes; a subset of these genes may play a role in survival and reproduction of female worms.

## Methods

### Ethics statement

All animals were handled in strict accordance with good animal practice as defined by the Animal Welfare Act, the Guide for the Care and use of Laboratory Animals and guidelines of the Division of Comparative Medicine, Washington University School of Medicine.

### Infections and parasites

Mongolian gerbils (*Meriones unguiculatus*) (Charles River Laboratories, Wilmington, MA) were inoculated subcutaneously (s.c.,) with 150 *B. malayi *L3 larvae as described previously [38]. Adult worms at 180 days post-infection were recovered from testis, spermatic cord lymphatics, heart and lungs. Female worms were separated from males, washed in RPMI-1640, and were snap frozen in dry ice/ethanol and stored at -80 C. All worms recovered from s.c., infections used in this study were of the same age group but derived from different gerbils.

### *In vitro *culture of female worms

*Brugia malayi *female worms from treated and untreated gerbils were cultured *in vitro *to assess microfilaria (MF) release, and the number of released MF were counted on days 2, 4, and 6 of culture [6]. Briefly, 5 worms were incubated per well in 6-well culture plates (Costar, Cambridge, MA) in 5 ml of complete medium (RPMI-1640 supplemented with 25 mM HEPES buffer, 2 mM glutamine and 10% fetal calf serum) at 37 C in a 95% air-5% CO_2 _atmosphere.

### Doxycycline treatment and worm recovery

Subcutaneously infected gerbils (n = 6) with circulating microfilaria at approximately 140 days post infection were treated by oral gavage with doxy (Sigma Chemical Company, St. Louis, MO) in distilled water at 100 mg/kg body weight, daily for 6 weeks. Controls were untreated. At the end of the treatment, animals were necropsied, and worms were removed from animal tissues.

### Microfilaria counts and embryograms

MF in 10 μl of tail-blood of treated and untreated animals were counted in duplicate after 1, 2, 4 and 6 weeks of treatment [39]. *Brugia malayi *female worms recovered after 6 weeks of doxy treatment and control worms were studied to produce embryograms as previously described [6]. Differences between embryograms of treated and control worms were analysed by One-way ANOVA.

### Electron microscopy

Female worms from treated and untreated gerbils were fixed in 100 mM phosphate with 2% paraformaldehyde and 2.5% glutaraldehyde and post-fixed in 1% osmium tetroxide in phosphate buffer. Sections were stained, dehydrated in ethanol and embedded in Eponate 812 (Ted Pella, Inc., Redding, CA). Ultrathin sections (70-90 nm) were stained with uranyl acetate and lead citrate and viewed with a JOEL 1200 transmission electron microscope [6].

### RNA isolation, cDNA probe synthesis, and microarray analysis

Two groups of 15 female worms each from doxy treated and untreated animals (biological replicates) were crushed, and total RNA was extracted separately using TRIzol (Invitrogen Corporation, Carlsbad, CA) and treated with DNase (Ambion, Austin, TX) as per the manufacturer's instructions. RNA integrity and quantity were measured with a Bioanalyzer-model 2100 (Agilent technologies Inc., Palo Alto, CA).

The Version-2 Filarial Microarray contains 18,104 oligonucleotide elements (65mer) that uniquely represent coding sequences for 15,412 *B. malayi *genes or predicted genes, 804 *Wolbachia *genes, 1,016 *Onchocerca volvulus *genes, and 872 *Wuchereria bancrofti *genes. A recent publication provides extensive annotation for this array [40]; see also http://www.nematode.net).

In a preliminary experiment, we synthesized cDNA from RNA samples using oligo(dT) primers and Superscript II Reverse Transcriptase (Gibco BRL, Gaithersburg, MD). Hybridizations, scanning and data analysis were performed as previously described [40,41]. Oligo(dT) priming has been the most widely used method for conversion of mRNA into cDNA through reverse transcription [42,43]. However, these experiments did not provide optimal hybridization signals for bacterial transcripts which lack polyA. Therefore, later experiments employed a random priming method for probe synthesis. Briefly, cDNA was synthesized from 2 μg of RNA using a Genisphere kit (Hatfield, PA) according to the manufacturer's instructions (http://www.genisphere.com/pdf/array900mpx_protocol_v06-22-04.pdf). In order to have technical replicates, cDNA probes were dye labeled separately with Cy5 and Cy3 as per the manufacturer's instructions (PerkinElmer Life and Analytical Sciences, Waltham, MA).

Dye-labeled cDNA probes were hybridized to duplicate 65 mer oligos on the V2 filarial array. Two biological replicates and two technical replicates provided 8 hybridizations for each filarial oligomer on the array. cDNA hybridizations were done overnight at 43 C with the 3DNA Array 900 detection system. The slides were washed, and scanning and gridding were performed with a ScanArray Express HT Scanner-v3.0 (Perkin Elmer, Boston, MA). Photomultiplier tube (PMT) values were set to 68 and 59 volts for Cy3 and Cy5, respectively. An additional scan was done for each slide with the PMT set for 56 and 48 volts. The scanned fluorescence intensity values were Lowess normalized and analyzed by Genespring v7.2 (Agilent) and Partek Genomics Suite software (Partek, St. Louis, MO). An average of four arrays for each condition were considered and *w*Bm and Bm elements were scored as "present" if the signal was ≥ 250 or if the signal to background ratio was ≥ 2. All microarray experiments were performed in agreement with the MIAME guidelines. The microarray data sets have been deposited into GEO (accession ID; GSE34976).

Genes with fold differences in expression equal to or greater than two fold and a confidence level of 99% (P < 0.01, Student's t-test) in pair-wise comparison between treated and control worms were considered to be differentially expressed.

### Gene expression analysis by quantitative reverse transcriptase polymerase chain reaction (qRT-PCR)

Known *w*Bm gene sequences for 16s rRNA (*w*Bm9003), cell cycle gene-*fts*Z (*w*Bm0602), surface protein-wsp (AJ252061), seven heme genes (*w*Bm0133, *w*Bm 0373, *w*Bm0777, *w*Bm0728, *w*Bm 0001, *w*Bm0709 and *w*Bm0719), and *B. malayi *sequences for sheath protein-shp1 (U43568), embryonic fatty acid binding protein-FABP1 (AF178439), and actin (XM_001895760), were used to amplify the corresponding transcripts in treated and control worm RNA by qRT-PCR. These experiments were performed twice with two batches of worms. In addition, a subset of differentially expressed *w*Bm and Bm genes from the microarray analysis was selected for assay confirmation studies by qRT-PCR. The expression profiles for bm.03044, BMX1802, BMX9848, BMX8408, BMX9336, BMX11510, BMX4544, bm.00532, bm.01661, BMX2449 genes affected after antibiotic treatment (both in our array study and in previous reports) were also analyzed by RT-PCR using 6 wks treated *vs*. untreated worm cDNA. Primer pairs were designed from sequences obtained from the *w*Bm genome http://tools.neb.com/wolbachia/) or from GenBank with Primer Express 3.0 software (Applied Biosystems, Foster city, CA). Sequence specific primer pairs were purchased from Integrated DNA Technologies, Coralville, IA. Briefly, cDNA was synthesized using 1 μg of total RNA, random primers, and SuperScript II reverse transcriptase (Invitrogen, Carlsbad, CA). qRT-PCR reactions were performed in duplicate in 96-well optical plates in a 25 μl reaction volume containing 1 ng of cDNA with individually optimized primer concentrations and SYBR green master mix (Applied Biosystems). To ensure that there was no genomic DNA contamination in cDNA and no primer dimer artifacts, reactions containing templates generated without reverse transcriptase enzyme were included as controls. Water was also tested in each reaction as a "no template" control (NTC). Reactions were conducted with an initial step of 2 min at 50 C and 10 min at 95 C, followed by 40 cycles of 15 s at 95 C and 1 min at 60 C. A melting curve analysis was performed at the end of each cycle to assess the amplification specificity. Cycle threshold (C_t) _values were obtained for the target and internal control gene. *Brugia malayi *histone gene (Pub_locus Bm1_49345) C_t _values were used as an internal control to normalize transcript levels present in different samples. Real-time PCR efficiency (E = 10 ^- (1/slope)^-1) was monitored by generating standard curves for Bm histone using log template concentration of cDNA from treated and untreated worms and found to be close to 100%. The relative quantitation method (2 ^-ΔΔCt^) was used to compare expression levels of *w*BM and Bm genes in different samples [44] (http://www3.appliedbiosystems.com/cms/groups/mcb_support/documents/generaldocuments/cms_040980.pdf).

For correlation analysis, qRT-PCR results (2^-ΔΔ ^C_t_) of selected *w*Bm and Bm genes and their corresponding microarray results (normalized ratios) were Log_10 _transformed, and the results were analyzed by the nonparametric Spearman rank correlation test using GraphPad Prism v.4 software (GraphPad Software Inc, San Diego, CA). qRT-PCR results for individual genes were analysed by one-way ANOVA to assess the significance of differences between treated and control worms.

### Functional classification of differentially expressed gene transcripts

Functional analysis was carried out for differentially expressed *w*Bm and Bm genes to identify overrepresented pathways using the Kyoto Encyclopedia of Genes and Genomes (KEGG v.43) [45]. For each query, the top match (with *P *< 1.0e-10) and all the matches within 30% of the top Blast score were accepted for KEGG Ontology (KO) and pathway associations. Default parameters for InterPro Scan v13.1 were used to map against InterPro database [46]. These mappings were used to classify the gene transcripts as biological, molecular, and cellular components. Gene Ontology mappings for *w*Bm genes were selected from UniProtKB database (http://www.uniprot.org/uniprot/) and from InterProScan v13.1. Statistically enriched pathways and gene ontology (GO) categories were obtained using hypergeometric analysis with *P *< 0.05 considered significant [47]. For non-redundant (NR) nucleotide hits, differentially expressed genes were BLAST searched (e-value cutoff of 1.0e-10) against a NR database with no *Caenorhabditis *sequences. In addition, we used Wormpep v.180 with an e-value cutoff of 1.0e-05 for detecting *Brugia malayi *homologs of *C. elegans *genes having RNAi phenotypes.

## Results

### Effect of doxycycline on *Brugia malayi *worms and *Wolbachia*

**Figure 1:**
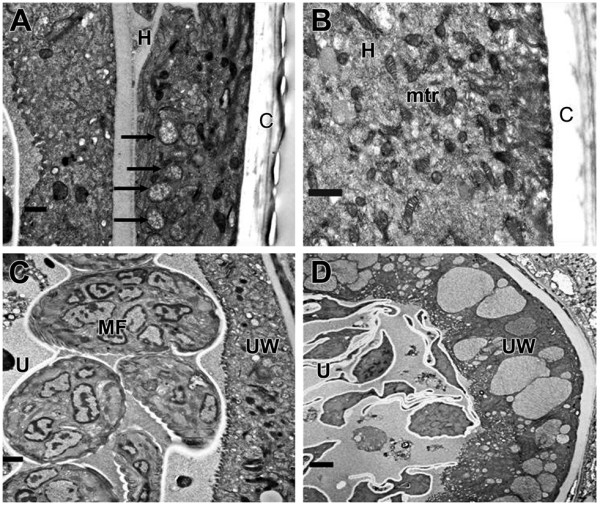
**Transmission electron micrographs of *Brugia malayi *female worms**. Panel A and C are sections of untreated female worm. Panel A shows numerous *Wolbachia *(arrows) in the hypodermis. Panel C shows, intact embryos in the uterus. Panel B and D are sections from doxycycline treated worms. The hypodermis is free of *Wolbachia *(Panel B) and only degenerate embryos were present in the uterus of treated worms (Panel D). U, uterus; C, cuticle; UW, uterine wall; MF, microfilaria; mtr, mitochondria. Scale bar = 1 μm.

Our aim was to study 6-week treatment effects of doxy on adult female worms and their *w*Bm. Doxycycline did not reduce circulating MF counts in gerbils (Table 1). Although there was some reduction (15%) in adult worm recovery in the treated group, this difference was not statistically significant. Interestingly, female worms recovered from treated gerbils did not release MF in vitro. In contrast, MF counts on days 2 and 4 of culture from untreated worms were 1.7 × 10^3 ^± 0.5 × 10^3^/ml and 2.1 × 10^3 ^± 0.9 × 10^3^/ml, respectively. Embryograms of treated worms were abnormal and different from those of control worms (see additional file 1). Late developmental stages (stretched MF and pretzels) were markedly reduced in treated worms relative to untreated worms. Ultrastructural studies clearly showed *w*Bm bacteria in the hypodermis of untreated worms but not in treated worms (Figure 1A, B). Degenerate worm embryos were observed in treated worms (Figure 1D) while intact embryos were present in controls (Figure 1C).

**Table 1:** In vivo effects of doxycycline on *Brugia malayi *microfilaria (MF) and adult worms

	Stage	% of pretreatment MF counts (mean ± SD)				Mean (± SD) number of worms (% reduction in worms)
		**Days after treatment**				

		7	14	28	42	42

Treated	MF	121 ± 74	119 ± 30	200 ± 95	192 ± 101	-
Control	MF	167 ± 135	90 ± 18	143 ± 108	112 ± 92	-
Treated	Adult worm	-	-	-	-	29.3 ± 18 (15)
Control	Adult worm	-	-	-	-	34.5 ± 10

Gerbils (n = 6) were treated with doxycycline (100 mg/kg/bw/per os). Controls were untreated. During 6 weeks of treatment, on day 7, 14, 28, and 42 no significant reduction in MF counts was observed. Reduction in worm recovery at the end of the treatment between control and treated gerbils was not significant.

### Effects of doxycycline treatment on *Wolbachia *gene expression

The V2 Filarial array contains 804 *w*Bm genes, and hybridization signals were detected for 679 (84.5%) in control female worm cDNA (see additional file 2). Expression signals were detected for 538 (67%) of *w*Bm genes in doxy treated worm RNA (18% fewer than in untreated worms).

Of 679 *w*Bm genes with detectable expression signals in control worms, the top twenty with the highest expression signals (median values 3.08E+03 to 1.02E+04) included genes involved in protein synthesis (6), stress response (4), antioxidant (1), energy and electron transfer (1), copper acquisition and aerobic respiration (1) lipid metabolism (2), DNA binding or repair (2), host interaction and adaptation (3) and hypothetical (4) (see additional file 3). Expression signals for 140 *w*Bm elements were present in control worms and absent in treated worms. The top 10 genes that showed highest expression signals in control worms but 'absent' in treated worms include molecular chaperonins, transcription and translation factors, membrane and ribosomal proteins and antioxidants (see additional file 4). In contrast, signals for 98 *w*Bm elements were present in treated worms but absent in control worms (see additional file 4). The top 10 genes with highest expression signals here include transport or lipoprotein release (*w*Bm 0483), secretion (*w*Bm 0796), proteolysis (*w*Bm 0770), carbohydrate and aminoacid metabolism (*w*Bm 0666), cell wall biogenesis and peptidoglycan biosynthesis (*w*Bm 0508), nucleic acid repair (*w*Bm 0746), and integral membrane protein TerC (*w*Bm 0442) which may be involved in resistance to antibiotics.

The scatter plots displaying the mean normalized fluorescence intensity signals for all elements on the array (panel A) and for *w*Bm elements alone (panel B) met the criteria for gene expression in treated vs. control samples (see additional file 5).

Of 538 *w*Bm genes with expression signals in treated worms, two hundred (37%) genes having signals in both treated and control worms showed differential expression (≥ 2, P ≤ 0.01), and all but 3 of these were down-regulated (see additional file 6). *w*Bm0474, which may be essential for electron transfer and energy metabolism, showed increased expression. Among the down-regulated genes 38 (19%) were ribosomal transcripts. The top 18 genes with known functions that showed significantly lower expression include transcripts involved in translation, DNA replication, recombination and repair, surface protein, Type IV secretory system, or nucleotide and nucleoside metabolic processes; these functions are associated with the cell nucleus, ribosome, cytoplasm or cell membrane (Table 2).

**Table 2:** Description of top 20 differentially expressed *Wolbachia *genes found in doxycycline treated *Brugia malayi *female worms

Functional class	Systematic	Annotation#	Fold change	P-value	Signal	Description
**Protein synthesis**						
	BMX14197	*w*Bm0431	0.12	1.84E-05	Down	DNA-directed RNA polymerase sigma 32 subunit, RpoH
	BMX14096	*w*Bm0328	0.11	4.19E-05	Down	Ribosomal protein S14
	BMX14288	*w*Bm0521	0.23	7.90E-05	Down	Ribosomal protein S21
	BMX14418	*w*Bm0650	0.12	9.18E-05	Down	Ribosomal protein L1
	BMX14100	*w*Bm0332	0.10	9.84E-05	Down	Ribosomal protein S17
	BMX14101	*w*Bm0334	0.19	1.01E-04	Down	Ribosomal protein L16
	BMX14113	*w*Bm0346	0.16	1.14E-04	Down	Ribosomal protein S12
	BMX14026	*w*Bm0258	0.21	5.92E-05	Down	Ribonuclease D
**DNA replication, DNA recombination, DNA repair or SOS response**						
	BMX14200	*w*Bm0434	0.06	1.56E-05	Down	DNA polymerase III, gamma/tau subunit
	BMX14193	*w*Bm0427	0.25	5.30E-05	Down	RecA recombinase
	*BMX14131*	*wBm0365*	*3.06*	*1.19E-03*	*Up*	*Predicted EndoIII-related endonuclease*
	BMX14476	*w*Bm0708	0.13	1.22E-04	Down	Superfamily II DNA/RNA helicase
**Energy production and conversion**						
	BMX14457	*w*Bm0689	0.09	8.65E-05	Down	F0F1-type ATP synthase, beta subunit
	*BMX14240*	*wBm0474*	*3.16*	*1.49E-03*	*Up*	*NADH:ubiquinone oxidoreductase*
**Metabolism & Transport**						
	BMX14214	*w*Bm0448	0.14	1.40E-04	Down	Succinate dehydrogenase flavoprotein subunit, SdhA
	BMX13916	*w*Bm0146	0.23	1.38E-04	Down	Kef-type K+ transport system, membrane component
	BMX13899	*w*Bm0129	0.27	1.09E-04	Down	Ornithine/acetylornithine aminotransferase
	BMX13796	*w*Bm0024	0.15	1.06E-05	Down	HesB/YadR/YfhF family protein
**Secretion**						
	BMX14563	*w*Bm0795	0.18	1.27E-04	Down	Type IV secretory pathway, VirB6 components
**Immunogenic membrane components**						
	BMX14198	*w*Bm0432	0.06	1.21E-04	Down	Outer surface protein-wsp

Expression of listed *Wolbachia *genes was down-regulated and those shown in *italics *were up-regulated after doxycycline treatment in vivo.

KEGG data search revealed that out of 804 *w*Bm genes on the microarray, 499 were mapped to many pathways ranked by gene abundance, and 487 had KEGG ontology (KO) assignments (see additional file 7). Many mapped genes were associated with pathways in metabolism (221), genetic information and processing (215), environmental information and processing (46) and cellular processes (13). Out of 200 *w*Bm genes that were differentially expressed after doxy treatment, 141 (70%) genes were mapped to pathways, and significantly enriched pathways (*P *≤ 0.05) associated with down-regulated genes include bacterial ribosome, translation factors, and the pentose phosphate pathway in carbohydrate metabolism (Table 2, see additional file 7). In contrast, two *w*Bm genes (*w*Bm 0474, *w*Bm 0365) with increased expression after treatment mapped to pathways involved in energy metabolism, nucleic acid replication/protein repair, respectively (see additional file 7). The third up-regulated gene (*w*Bm0149) is a hypothetical gene and its function is unknown. GO term assignments were concordant with KEGG results, and many biological processes and molecular functions in *w*Bm were down-regulated after doxy treatment (see additional file 6). InterPro scans showed a total of 366 protein domains mapped in *w*Bm gene transcripts, and only two of these domains related to nucleic acid binding and to an unknown function were significantly (*P *< 0.05) enriched in down-regulated *w*Bm genes (see additional file 8). The protein domains mapped to the up-regulated *w*Bm 0474, *w*Bm 0365 genes are related to the respiratory chain proteins NADH-quinone oxidoreductase and dehydrogenease subunits and DNA repair proteins.

### Effect of doxycycline treatment on *Brugia malayi *(Bm) gene expression

Of 15,412 Bm elements on the array, 13,399 had hybridization signals in control adult female worms. Signals were detected for 14,340 elements in doxy treated worm cDNA (see additional file 2). 53 Bm elements were present in control worms and absent in treated worms. In contrast, 994 Bm elements were present in treated but absent in control worms. Of 53 elements uniquely present in control worms, elements for 15 genes have no annotation in the database and 9 were hypothetical (see additional file 9). Known genes included cytoskeletal genes (actin, myosin), the antioxidant glutathione S-transferase (GST), and ribosomal proteins (S1 and L11). KEGG analysis showed pathways associated with metabolism (biosynthesis of secondary metabolites, xenobiotic metabolism and degradation, and glycan biosynthesis), cellular processes (cell motility and communication, and immune system), and human diseases were enriched significantly (*P *≤ 0.05) in control worms (see additional file 10). Of 994 genes with expression signals in treated worms but not in control worms, many were classified as hypothetical (288), ribosomal (8) and novel genes (~480) with no annotation. Known genes in this group included cuticular components, proteases (astacin and ADAMTS-like metalloproteases, cathepsin-L (cpl-1), vespid allergen antigen homolog (Val), immunogens and immunomodulators (Alt-1) (see additional file 9). KEGG pathways significantly (*P *≤ 0.05) enriched in treated worms included metabolism of carbohydrates and secondary metabolites, energy metabolism (oxidative phosphorylation), and nucleotide metabolism. In addition, pathways involved in genetic information and processing (translation, folding), signaling pathways and various other cellular processes (cell motility, cell growth and communication) were enriched in treated worms (see additional file 10). Many GO terms assigned for genes present in control worms and treated worms or vice versa corresponded to pathways analysed by KEGG, and only those significantly (*P *< 0.05) enriched are listed in additional file 10.

546 Bm genes present in both control and doxy treated worms were differentially expressed after doxy treatment (see additional file 2). Of these, 462 were up-regulated including those that encode genes associated with energy metabolism, electron transport, antioxidants, antigenicity, immuno-modulation/evasion, and many novel or predicted genes with unknown biological functions (Table 3; see additional file 11). Interestingly, we observed that many mitochondrial transcripts with oxidoreductase activity (14981.m02381, 14243.m00195, 14972.m07606, 13478.m00071, 14773.m00936, BMC11992) were significantly up-regulated. In addition, mitochondrial transcripts of complex II (cytochrome c-oxidase) and complex III (ubiquinol-cytochrome c-oxidase) subunit which are associated with oxidative phosphorylation in energy metabolism and electron transfer were significantly up-regulated, whereas NADH dehydrogenase subunit-5 was down-regulated. The 84 (16%) Bm genes that were down-regulated after treatment included many known genes that encode cuticular components (collagen, caveolin and cadherin), embryonic fatty acid binding protein, and novel genes with unknown functions (see additional file 11). Of 14,340 elements with hybridization signals in treated worms (see additional file 2), signals for 34 heat shock associated transcripts were detected including small heat shock proteins, Hsp-90, and Hsp-70 in treated and untreated worms (data not shown). However, these were not differentially expressed.

**Table 3:** Major known differentially expressed* *Brugia malayi *genes after doxycycline treatment

Functional class	Sequence	OligoID	Fold change	*P *value	Description	Pub_Locus
**Up-regulated**						
Electron transfer						
	BMC05513	bm.01224	5.12	1.04E-03	Cytochrome C oxidase	Bm1_53760
	14963.m01788	BMX5456	2.66	3.13E-03	Ubiquinol-cytochrome-c oxidase activity	Bm1_34585
	BMC01497	bm.00393	3.55	6.56E-03	ATP synthase F0 subunit 6	
Antioxidants						
	14981.m02381	BMX7492	2.85	1.72E-03	Thioredoxin	Bm1_46710
Proteases						
	BMW00062.466	BMX11239	3.12	1.42E-03	Cathepsin L-like cysteine proteinase	Bm1_00065
	13332.m00193	BMX1173	2.65	4.18E-03	Metalloprotease	Bm1_07750
	13632.m00184	BMX1614	2.01	7.91E-03	Trypsin family protein	Bm1_10665
Metabolism and transport						
	14773.m00936	BMX4045	2.67	5.91E-03	Pyruvate dehydrogenase	Bm1_25865
	13478.m00071	BMX1447	2.00	5.55E-03	Malate/L-lactate dehydrogenase	Bm1_09530
	14981.m02410	BMX7517	3.61	3.27E-04	Biopterin-dependent aromatic amino acid hydroxylase	Bm1_46865
	14787.m00179	BMX4095	13.7	7.23E-06	Cysteine-rich, acidic integral membrane protein precursor, putative	Bm1_15145
Immunogens						
	14222.m00064	BMX2433	10.3	2.76E-05	PPE family protein, putative	Bm1_15875
	BMC05381	bm.01203	2.13	4.10E-03	BmSERPIN	Bm1_03995
	12920.m00009	BMX601	2.49	6.84E-03	Excretory/secretory protein Juv-p120	Bm1_04050
	BMC00351	bm.03044	2.27	2.65E-03	Vespid venom allergen antigen-like protein 1	Bm1_14040
	14399.m00013	BMX2941	2.51	7.90E-04	ALT protein	Bm1_14360
	13893.m00088	BMX1920	4.25	3.08E-03	IgG and IgE immunoreactive antigen	Bm1_12575
	BMC00480	bm.00167	4.40	2.76E-03	mmc1	#N/A
	13673.m00036	BMX1683	3.03	1.05E-03	Mucin	Bm1_11110
**Down-regulated**						
Reproduction, development & transport						
	BMC00903	bm.00277	0.47	2.78E-03	Embryonic fatty acid-binding protein Bm-FAB-1	Bm1_33050
	12495.m00012	BMX108	0.26	8.99E-03	Collagen, putative	Bm1_00775
	15377.m00007	BMX9051	0.21	1.75E-03	Alpha-1 collagen type IX, putative	Bm1_56350
	AA109462	BMX9336	0.40	5.12E-03	Peptidyl-prolyl cis-trans isomerase	Bm1_56870
	BMC03244	bm.00803	0.45	4.98E-03	Prolyl 4-hydroxylase	Bm1_45455
	BMC02383	bm.00595	0.44	6.93E-04	Caveolin-1	Bm1_36280
	14700.m00144	BMX3736	0.42	4.38E-03	Cadherin	Bm1_24010
Electron transfer						
	BMC02188	bm.00554	0.30	2.20E-03	NADH dehydrogenase subunit 5	Bm1_56750

### KEGG and InterPro analysis of differentially expressed *B. malayi *genes

**Figure 2:**
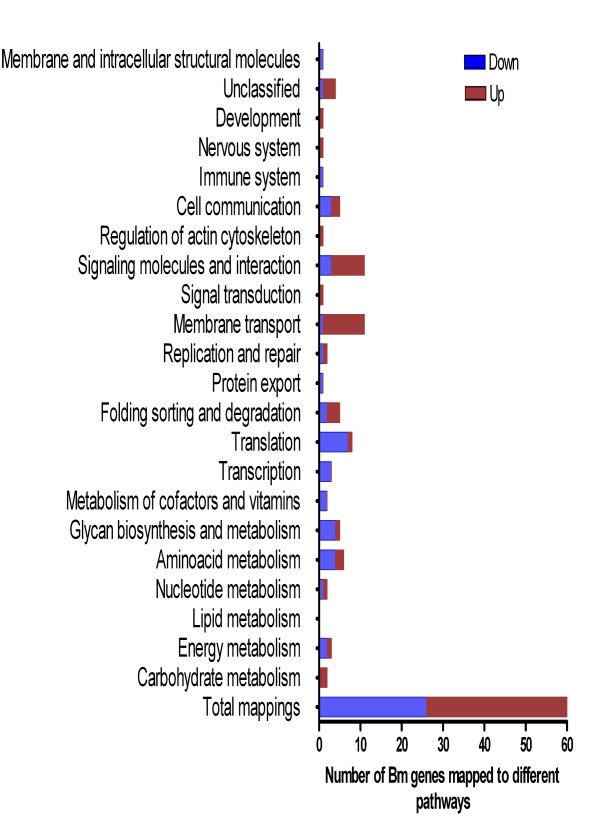
**Distribution of KEGG pathways mapped to up-regulated and down-regulated *Brugia malayi *genes after doxycycline treatment**. Many genes involved in transport, signaling, tanslation, metabolism and cell communication pathways were affected. Significant canonical pathways (*P *≤ 0.05) and number of genes mapped in each pathway and their percentages are shown in additional file 12 for regulated genes.

KEGG-based analysis mapped 60 Bm genes that were differentially expressed after doxy treatment to different pathways. Several of these were grouped to environmental information and processing, genetic information and processing, metabolism and transport (Figure. 2, see additional file 11, 12). Transport and signaling pathways significantly enriched (*P *≤ 0.05) were mapped to up-regulated genes. Significant pathways mapped to down-regulated Bm genes included amino acid metabolism, cellular processes and ribosomal functions (see additional file 12, Figure 2). InterPro analysis showed that a large number of up-regulated genes contained zinc finger domains and retroelements (see additional file 13). Other protein domains associated with integration of retroelement cDNA into host genome (integrase, polynucleotidyl transferase) were also enriched in up-regulated genes. Other domains in this group were associated with immune evasion (ALT), proteases (aspartic acid proteases) and energy metabolism (glyceraldehyde 3-phosphate dehydrogenase). Enriched protein domains mapped to the down-regulated genes included ribosome, cytoskeleton and bacterial protein transport. The complete list of significant protein domains is shown in additional file 11, 13. DNA integration (GO: 0015074), proton (GO:0015992), electron (GO:000618) and ion transport (GO:0006813), signaling cascades (GO:0007242, GO:0007186), glycolysis (GO:0006096) were some of the highly represented biological function ontologies linked to up-regulated Bm transcripts. Phosphate transport (GO:0006817), regulation of translation and transcription (GO:0006350, GO:0045449, GO:0006414) and protein biosynthesis (GO:0006412) and transport (GO:0015031) were highly represented biological ontologies to down-regulated Bm transcripts. The other major molecular functions and cellular components enriched in GO are listed in additional file 11.

### Comparison of observed changes in *Brugia malayi *and *Litomosoides sigmodontis *gene expression after doxycycline/tetracycline treatment

We compared our results with those recently reported after i.p. tetracycline treatment of Bm and in *L. sigmodontis *[48,49]. We have observed expression of more genes to be regulated after doxy treatment in Bm compared to the two previous reports (319 and 323 *vs *546 respectively). However, only few genes (1% or 5/546) regulated at 6 wks doxy treatment agreed with differentially expressed genes found in Bm or *L. sigmodontis *at earlier time points. The expression of vespid venom allergen homolog (BMC00351) while it was down- regulated in Bm at 2 wks of treatment, found up-regulated at 5 and 6 wks in *L. sigmodontis *and Bm. Genes encoding hypothetical (BMX1802, BMX9848), trafficking protein (BMX 8408) and peptidyl-prolyl cis-trans isomerase (PPI-FKBP type, BMX9336) transcripts were up-regulated at 2 wks but down-regulated at 6 wks of treatment in Bm. In addition, the Bm homologs of seven-transmembrane-helix (7TM) receptor (BMX11510), zinc finger family protein (BMX4544) and 2 hypothetical proteins (bm.00532, bm.01661) found in *L. sigmodontis *were in agreement with regulated (up) genes at 6 wks. However, expression of nucleotide-binding protein (BMX2449) involved in signaling pathway was down in *L. sigmodontis *at 5 wks but up at 6 wks in Bm. Our microarray results for these genes were further confirmed by RT-PCR (data not shown).

### Homologs of *Caenorhabditis elegans *genes with known RNAi phenotypes in doxycycline treated *B. malayi*

A total of 321 genes of the 994 Bm elements present in treated female worms and not in control worms have homologues in *C. elegans *with RNAi phenotype information. While 228 genes were wild type phenotypes, 93 had abnormal phenotypes. Most of these are involved in reproduction and development including 50 embryonic lethal (Emb), 26 growth (Gro), 35 sterility (Ste) and sterile progeny (Stp) phenotypes (see additional file 9).

272 of 546 (50%) Bm transcripts that were present in both control and treated worms but differentially expressed had homologues in *C. elegans *(see additional file 11). More of the genes that were up-regulated after doxy (231/272) had homologues than genes that were down-regulated (41/272). RNAi phenotype information was available for *C. elegans *homologues for 264 of 546 (48%) Bm genes. Of these, 207 homologues were wild type, while 57 (36 up-regulated and 21 down-regulated genes) had severe phenotypes in *C. elegans *including sterility, larval arrest, embryonic lethality, egg laying defect, sick, slow growth, *etc*. In addition, 19 down-regulated Bm genes with observed phenotypes have been reported to be female-regulated (Li et al, personal communication). These included four collagen genes (15377.m00007, 14845.m00009, 14296.m00065, 14296.m00063), one histone (13785.m00207), and one hypothetical gene (BMC01609). Many other down-regulated Bm genes are homologues of sterile phenotypes in *C. elegans *especially Emb, Lva, Lvl, Ste phenotypes.

### Gene expression analysis by qRT-PCR

**Figure 3:**
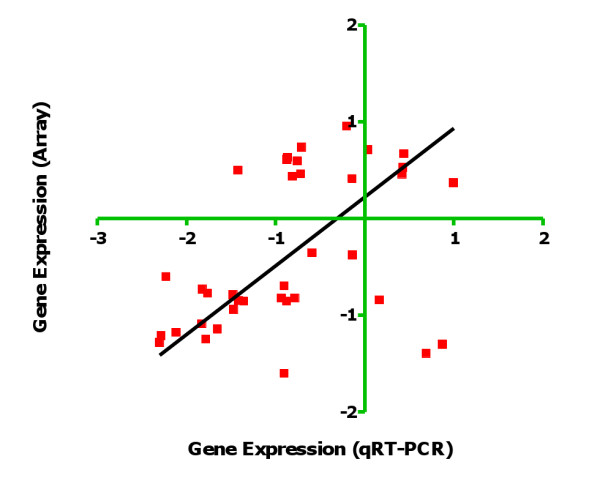
**Correlation between expression values for *Wolbachia *and *Brugia malayi *genes obtained by microarray (y-axis) and qRT-PCR (x-axis)**. Normalized fold change values of microarray experiment and 2^-ΔΔ Ct ^values of qRT-PCR obtained for the same genes were Log transformed and analysed by the nonparametric Spearman rank correlation test (R = 0.4352; *P *= 0.007).

qRT-PCR studies were performed for 37 genes with significantly different expression signals between treated and control worms by microarray. These included 25 Bm genes and 12 *w*Bm genes. qRT-PCR results were consistent with array results for 25 of 37 genes (68%), and quantitative results obtained with these two methods were significantly correlated (R = 0.4352; *P *= 0.007) (Figure 3). In some cases without agreement, differences by qRT-PCR were in the same direction as those observed by microarray, but the qRT-PCR differences were not statistically significant.

qRT-PCR studies showed significant down-regulation of *w*Bm 16S, *fts*Z and wsp expression in doxy treated female worms relative to controls. Down- regulation was more impressive for 16S and wsp genes compared to *fts*Z (see additional file 14). Embryo associated genes Bm-shp1 and FABP transcripts were significantly (*P *< 0.05) down-regulated in treated worms, while treatment did not significantly affect actin expression. Interestingly, expression of all 7 *w*Bm genes in the heme synthesis pathway was significantly down- regulated in treated worms by qRT-PCR (see additional file 14). Again, these results were generally consistent with those obtained by microarray. Two genes (*w*Bm 0709 and *w*Bm 0719) had no expression signal by microarray in treated worms. Expression signals for the other 5 genes were decreased in treated worms, although only one of these (*w*Bm 0133, 5-aminolevulinate synthase, EC 2.3.1.37) met the criterion of a 2-fold reduction with *P *< 0.01 (see additional file 3).

## Discussion

Prior studies have shown that doxy affects *w*Bm from filarial worms with disruption of parasite development and reproduction. Ultrastructural changes and changes in cell- surface (wsp) and cell- cycle (*fts*-z) DNA markers relative to controls indicate that doxy treatment reduces *w*Bm bacteria in female worms. The purpose of this study was to examine the effects of doxy treatment on gene expression in adult worms and in *w*Bm. As expected, our results confirmed previous reports regarding the effects of doxy treatment on *w*Bm, embryogenesis and MF production in adult filarial worms [6,13,15,16,23,24,50,51]. Although there are reports that doxy kills adult worms in some in vivo systems, our results showed that *Brugia malayi *worms survived 6 wks of doxy treatment in gerbils. Surviving worms were not normal, and a longer post-treatment observation period might have shown that doxy shortened the lifespan of adult female worms.

We then turned our attention to gene expression. We first tested the efficiency of cDNA probes synthesized by oligo(dT) priming for hybridization and signal detection. This method detected expression signals in adult female worms for 12,791 of 18,101 elements on the array (data not shown). However, this method was not reliable for detecting *w*Bm genes, which lack poly-A tails. Oligo(dT) primers also have a tendency to produce truncated cDNAs through internal poly(A) priming, and this can reduce the quality of synthesized cDNA [42,52]. Random priming of total RNA for synthesis of hybridization probes [53,54] worked well for transcripts of both *w*Bm and Bm. Untreated control female worms had transcription signals for 84% of *w*Bm genes, with most genes showing ≥ 3.08 × 10^3 ^fluorescence units. Presumably some of these *w*Bm genes are critical for bacterial survival and for maintenance of the mutualistic relationship association with their filarial host. Many *w*Bm gene expression signals were absent or significantly reduced after doxy treatment [55]. This finding was expected as doxy treatment kills and clears *w*Bm from filarial worms. It is unclear why three *w*Bm genes had increased expression signals after doxy treatment; *w*Bm0474, associated with energy metabolism and metabolism of cofactor molecules and *w*Bm0365 which is involved in DNA replication and repair were up-regulated. Similarly, in other studies of gene expression in tetracycline treated worms, expression of many *w*Bm genes was differentially expressed with 13 genes in *B. malayi *(at day 14) and 3 genes in *L. sigmodontis *(at day 36) up-regulated [48,49]. These results suggest that some *w*Bm and *w*Bm DNA persists after at least 6 wks of doxy treatment.

We found over representation of *w*Bm genes in pathways such as carbohydrate metabolism, energy metabolism, aminoacid metabolism and nucleotide metabolism that were affected by doxycycline treatment. Complete pathways for *de novo *biosynthesis of nucleotide/nucleoside biosynthetic genes are present in the *w*Bm genome, and this has been postulated as a basis for *Wolbachia *dependence in filarial worms [29]. Similarly, computational predictions suggested that *w*Bm genes (~552 genes) may be essential for certain pathways [56]. We observed that many genes predicted to have these functions were down regulated after doxy treatment.

Two recent reports described effects of tetracycline treatment on gene expression in Bm and *L. sigmodontis *[48,49]. Our study confirmed some of these observations (down-regulation of transcripts essential for transcription, translation), but there were also important differences (e.g., reduced expression of genes involved in reproduction and development, see Table 3). For example, Ghedin et al., [48] reported overexpression of Bm transcripts involved in amino acid synthesis and protein translation and down-regulation of cuticle synthesis transcripts very soon after tetracycline treatment (14 days). Similarly, while many transcripts involved in transcription and translation, and in metabolic and signaling pathways were down regulated, motility, heme-binding protein, and mitochondrial transcripts encoding energy metabolism were up-regulated after treatment in *Litomosoides *[49], and a similar profile for energy genes was reported for Bm [48].

In addition to the timing of gene expression changes in filarial worms, the experimental design and data analysis may explain differences in microarray results in the present study from those reported in prior studies. For example, the infection method (i.p., *vs*. s.c.,), medication used, mode and duration of drug treatment, and methods for probe synthesis and gene expression analysis in the present study differed in many ways from those used in the other reports. It is difficult to quantify drug dosage when medications are provided when it is in drinking water, and drugs can have different effects on worms in different locations in the host. In addition, we have used a more stringent criterion for calling differential expression by using > 2 fold change with P < 0.01 value as a parameter. We think this increased stringency is important to reduce false calls for differential gene expression in large microarrays.

Our study found striking increases after treatment in expression of genes encoding proteins involved in immunoregulation, electron transfer and energy metabolism. Many immune evasion gene transcripts were highly expressed in worms (Table 3) [57]. In addition, we found that *Brugia *mmc1 transcript (reported to be specific for MF stage, see [58], an immunogen was up-regulated in doxy treated female worms by microarray, and this result was confirmed by qRT-PCR. Activation of immune evasion functions may be needed for treated worms to survive, because the loss of *w*Bm might otherwise render the worms more susceptible to host immune responses. Up-regulation of mitochondrial genes involved in electron transfer and respiratory chain after treatment suggests that doxy treatment may directly affect targets other than *w*Bm such as worm mitochondria [33]. This increased expression of mitochondrial transcripts after doxy treatment may partially protect filarial worms from the loss of *w*Bm so that they can survive. Interestingly, tetracycline has no direct effect on *Wolbachia*-free *A. viteae *worms or their mitochondrial respiratory chain transcripts [49]. Additional research will be needed to test the hypothesis that mitochondria and *w*Bm may have overlapping or complimentary roles in Bm worms.

Heme is an essential factor for development, survival and fertility of many nematodes. The *w*Bm genome contains all but one of the genes required for heme synthesis [29,30]. In contrast, only 1 gene in the heme biosynthetic pathway is present in the *B. malayi *genome (ferrochelatase, EC 4.99.1.1, Bm1_14320). This led to the hypothesis that filarial worms may depend on *w*Bm for heme synthesis. Recent studies that showed that inhibitors of heme synthesis by *Wolbachia *reduced worm viability support this hypothesis [59]. Our qRT-PCR studies found decreased expression of all *w*Bm genes involved in heme synthesis in doxy-treated treated female worms. Microarray results were similar, but less impressive. Recently, Strübing et al., [49] postulated that up-regulation of mitochondrial genes was essential for worms to maintain mitochondrial heme-dependent respiratory chain protein complexes for energy and homeostasis without *Wolbachia*. Our results support this hypothesis.

Many previous studies have reported changes in filarial worm embryogenesis and development after doxy treatment. *Wolbachia *is known to affect host oogenesis in insects, possibly by affecting expression of genes involved in this process [60,61]. We observed that after the loss of *w*Bm, many known Bm gene transcripts essential for cytoskeleton and extracellular matrix synthesis (prolyl 4-hydroxylase, collagen/cuticulin, laminin, and caveolin) and transcripts believed to be involved in embryogenesis and reproduction (embryonic fatty acid binding protein and cathepsin-L) were down-regulated [62-66]. Although, phosphate permease family gene (*Ls-ppe-1*) thought to be involved in nucleotide metabolism was up-regulated after tetracycline treatment, orthologues in Bm were not up-regulated at 6 wks of treatment. However, other phosphate transport genes in Bm were down-regulated after doxy treatment. This suggests that doxy may significantly affect phosphate transport to Bm and that dysregulation of phosphate and nucleotide metabolism could impair Bm embryo development. Down-regulation of development-associated transcripts like FABP1 and shp1 after doxy by qRT-PCR further supports the array results. Similarly, Ghedin et al. [48] showed down-regulation of few collagen genes after tetracycline treatment in Bm. Conversely many other collagen collagen genes up-regulated in treated *Litomosoides*. While other studies reported that expression of troponins and several collagens was up-regulated in treated *Litomosoides *and Bm worms [49,67], our results did not confirm these findings.

Zinc-finger domain genes have diverse roles in transcriptional modulation, translational control, protein degradation pathways, and growth regulation; and they are believed to have diverse, important roles in filarial biology [36,68]. We found that doxy treatment increased expression of these genes in Bm suggesting that protein-nucleic acid and protein-protein interactions may be increased in treated worms after the depletion of *w*Bm. Based on NR best hits in the protein sequences, transcripts encoding at least 10 Bm genes that were differentially expressed after doxy have strong homology to mosquito (*Anopheles gambiae*) transcripts involved in DNA integration (14927.m00099), nucleic acid binding (15547.m00008), citric acid cycle (13478.m00071) or transport (15547.m00008, 14979.m04385, 12526.m00074). Sequence similarities to mosquito gene fragments have been reported in the *Bm *genome and in V2 gene expression studies [36]; (Li et al., unpublished observations) and in proteomic profiles of tetracycline treated worms [67] indicating occurrence of DNA integration of mosquitoes with *Brugia *nematodes.

In *C. elegans*, RNAi screening has demonstrated abnormal phenotypes for about 5000 genes which is about 25% of all predicted genes [69,70]. It has been shown that about 30% of predicted genes in Bm with homologues in *C. elegans *have RNAi phenotypes in that species [36,37]. Among the genes represented in the V2 filarial array, approximately 37% with homologues in *C. elegans *have RNAi phenotypes [40]. The increased rate of phenotypes for filarial genes with *C. elegans *homologues suggest that many of these genes are necessary for core nematode pathways that are conserved across the phylum.

## Conclusions

In summary, this study has provided detailed information on effects of doxy treatment on gene expression in *w*Bm and adult female Bm. As expected, treatment generally reduced *w*Bm gene expression. However, this was not uniform, and expression of many *w*Bm genes was not affected by doxy. Doxycycline had a mixed effect on Bm gene expression. Reduced expression of genes involved in reproduction, growth and development was consistent with sterility observed in doxy treated worms. The up-regulated genes and pathways in treated worms were less predictable. Up-regulation of energy production (oxidative phosphorylation, carbohydrate metabolism), electron transport, antioxidants, proteases and immunomodulatory genes may represent parasite adaptations that permit survival (albeit with sterility) when related functions normally provided by *w*Bm are disrupted by doxy treatment. For example, the energy and electron transport findings suggest that increased mitochondrial activity may help the worms survive the loss of *w*Bm. It is possible that *w*Bm normally function as accessory mitochondria providing extra energy in metabolically active tissues. Additional work will be needed to test this interesting hypothesis. The challenge remains to determine which of these changes in gene expression are critical adaptations and which are secondary effects or general responses to stress conditions. It would be interesting to compare doxy effects on gene expression in filarial worms to those caused by other antifilarial drugs.

## List of abbreviations used

Bm: *Brugia malayi*; *w*Bm: *Wolbachia*; Doxy: doxycycline; qRT-PCR: quantitative reverse transcription polymerase chain reaction; KEGG: Koyoto Encyclopedia of Genes and Genomes; KO: KEGG Ontology; GO: Gene Ontology; RNAi: RNA interference; MF: microfilaria

## Competing interests

The authors declare that they have no competing interests.

## Authors' contributions

RR and GW conceived and designed the experiments. RR, YH and MH performed the experiments. RR, GW, SC, MH, SA and MM analysed the data and interpreted the results. RR and GW prepared the manuscript. All authors read and approved the final manuscript.

## Supplementary Material

Additional file 1 **Effect of doxycycline on *Brugia malayi *female worm embryogenesis**. Embryograms for untreated and doxycycline (Doxy) treated female worms. Data shown are percentages of early developing embryos, pretzels and stretched MF (microfilaria) in worm homogenates. Differences between embryograms from untreated and treated worms were highly significant (*P *< 0.001). Click here for file

Additional file 2 Distribution of *Wolbachia *and *Brugia malayi *elements in V2 filarial array hybridized with cDNA from doxycycline* treated or control worms. Click here for file

Additional file 3 ***Wolbachia *elements and their signal strength in V2 filarial array hybridized to control *Brugia malayi *worm RNA**. This table describes the baseline expression signals for top 25 *Wolbachia *genes and their predicted gene ontology (GO) annotations. Click here for file

Additional file 4 ***Wolbachia *genes in treated and control *Brugia malayi *worms**. This list shows *Wolbachia *elements with signals present (P) or absent (A) in treated and control worms Click here for file

Additional file 5 **Expression profiles for filarial and *Wolbachia *genes using the V2 filarial array after doxycycline treatment**. Expression signals were documented by plotting the photomultiplier (PMT) signals at high intensities. Panel A (for all elements on the array) and panel B (*Wolbachia *elements only) are scatter plots displaying the mean normalized fluorescence intensity signals for elements in treated *vs*. control female worms. Up-regulated and down-regulated genes are shown in red and blue, respectively. 200 and 546 genes showing ≥ 2 fold change with *P *≤ 0.01 were considered to be differentially expressed in *Wolbachia and Brugia malayi *after doxy treatment. Tx: treated; C: control. Click here for file

Additional file 6 **Differentially expressed *Wolbachia *genes in *Brugia malayi *(≥ 2 fold change, *P *< 0.01) after doxycycline treatment**. This table contains lists of up-regulated (in red) and down-regulated (in blue) *w*Bm genes with their normalized expression units (ratios and *P *values), gene descriptions, Basic Local Alignment Search Tool (BLAST)-NR hits and their available GO assignments. Click here for file

Additional file 7 **Functional classification by Koyoto Encyclopedia of Genes and Genomes (KEGG) of differentially expressed *Wolbachia *genes in *Brugia malayi *after doxycycline treatment**. Summary sheet include number of *w*Bm genes that are represented in the V2-filarial array and their associated pathways. Frequent pathways mapped to the differentially expressed *w*Bm genes are shown in this table with their ko numbers. A hypergeometric cumulative distribution function (cdf) for each KEGG pathway was applied to identify the enriched pathways. Statistically significant (*P *< 0.05) pathways ranked by gene abundance are shown in red. Click here for file

Additional file 8 **Results of InterPro scans to show protein domains enriched in the differentially expressed *Wolbachia *genes in *Brugia malayi *after doxycycline treatment**. Significantly enriched protein domains in *w*Bm are shown in red. Click here for file

Additional file 9 ***Brugia malayi *genes in doxycycline treated and control worms**. This list shows *Brugia malayi *elements with signals present (P) or absent (A) in treated and control worms. *C. elegans *homologues for RNAi phenotypes for corresponding Bm genes in treated worms are listed in this table. Click here for file

Additional file 10 **Functional analysis (KEGG and GO) for genes present in treated but absent in control *Brugia malayi *female worms**. Statistically significant pathways and GO terms are shown in this table. Click here for file

Additional file 11 **Summary of differentially expressed *Brugia malayi *genes after doxycycline treatment**. This table contains lists of up-regulated (in red) and down-regulated (in blue) genes, normalized expression units (ratios and *P *values) and their corresponding annotations, *Caenorhabditis elegans *Blast Hits, matching *C. elegans *RNAi phenotypes, GO assignments, mapped KEGG pathways and their KO hits. InterPro scan results for matched protein domains are included for many differentially expressed genes. Click here for file

Additional file 12 **Functional classification by KEGG of differentially expressed *Brugia malayi *genes after doxycycline treatment**. This table shows summary of KEGG mappings for all Bm genes represented in V2-filarial array and for differentially expressed Bm genes. Pathways ranked by gene abundance are shown for all regulated genes. Click here for file

Additional file 13 **Results of InterPro scan analysis for protein domains enriched in the differentially expressed *Brugia malayi *genes after doxycycline treatment**. Significantly (*P *≤ 0.05) enriched protein domains mapped to the Bm genes are shown in red. Click here for file

Additional file 14 **Results of qRT-PCR assays for *Wolbachia *and *Brugia malayi *genes in female worms after doxycycline treatment relative to control worms**. Panel A shows expression changes for *w*Bm 16S rRNA, *fts*Z and wsp transcripts and Bm-shp1, Bm-FABP1 and Bm-actin transcripts. Panel B shows changes in *Wolbachia *genes in the heme synthesis pathway. Expression profiles of 16S rRNA, *fts*Z and wsp and Bm-shp1, FABP and heme genes relative to controls were significantly down-regulated (*P *< 0.05). Click here for file
