# Supplementary figures and images for: Effects of Doxycycline on gene expression in Wolbachia and Brugia malayi adult female worms in vivo
Source: J Biomed Sci. 2012 Feb 9;19(1):21. doi: 10.1186/1423-0127-19-21 (PMC3352068; doi:10.1186/1423-0127-19-21)

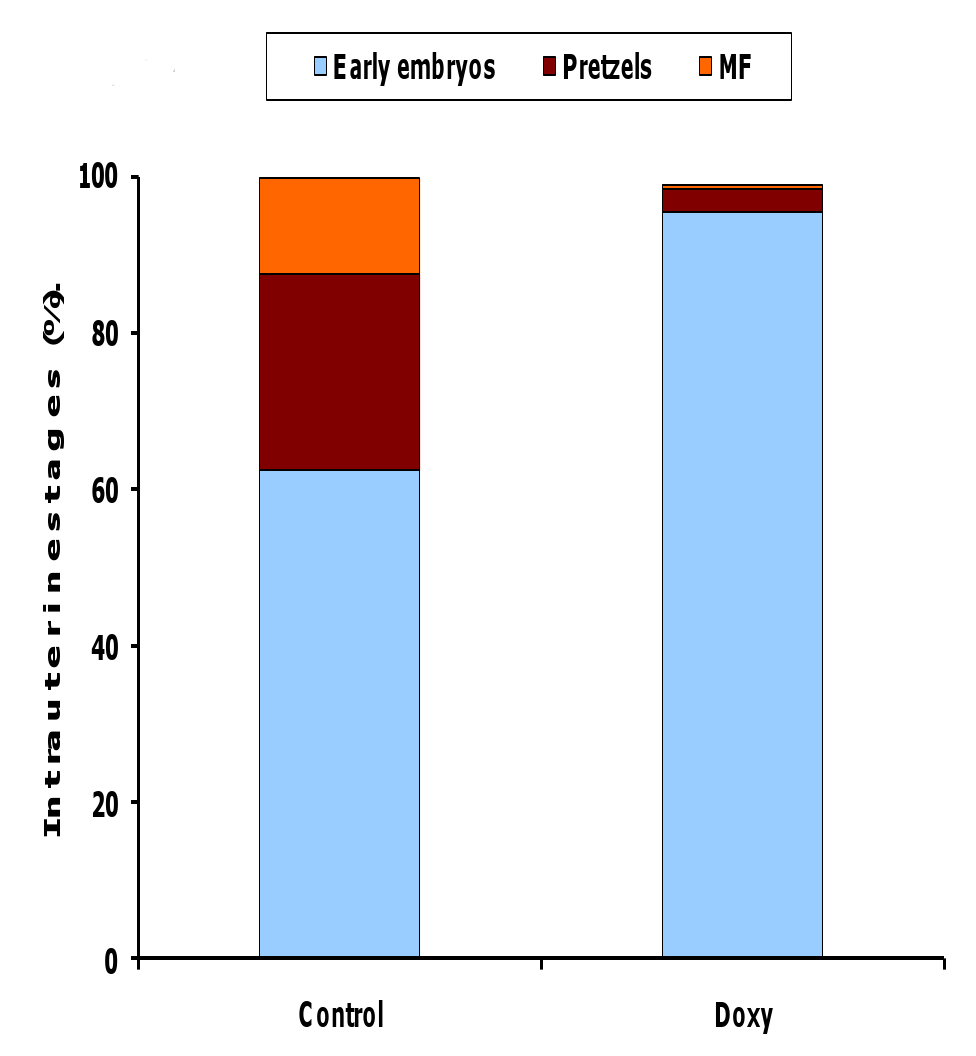

Supplement: Additional file 1 — Effect of doxycycline on Brugia malayi female worm embryogenesis. Embryograms for untreated and doxycycline (Doxy) treated female worms. Data shown are percentages of early developing embryos, pretzels and stretched MF (microfilaria) in worm homogenates. Differences between embryograms from untreated and treated worms were highly significant (P < 0.001). [file 1423-0127-19-21-S1.TIFF]

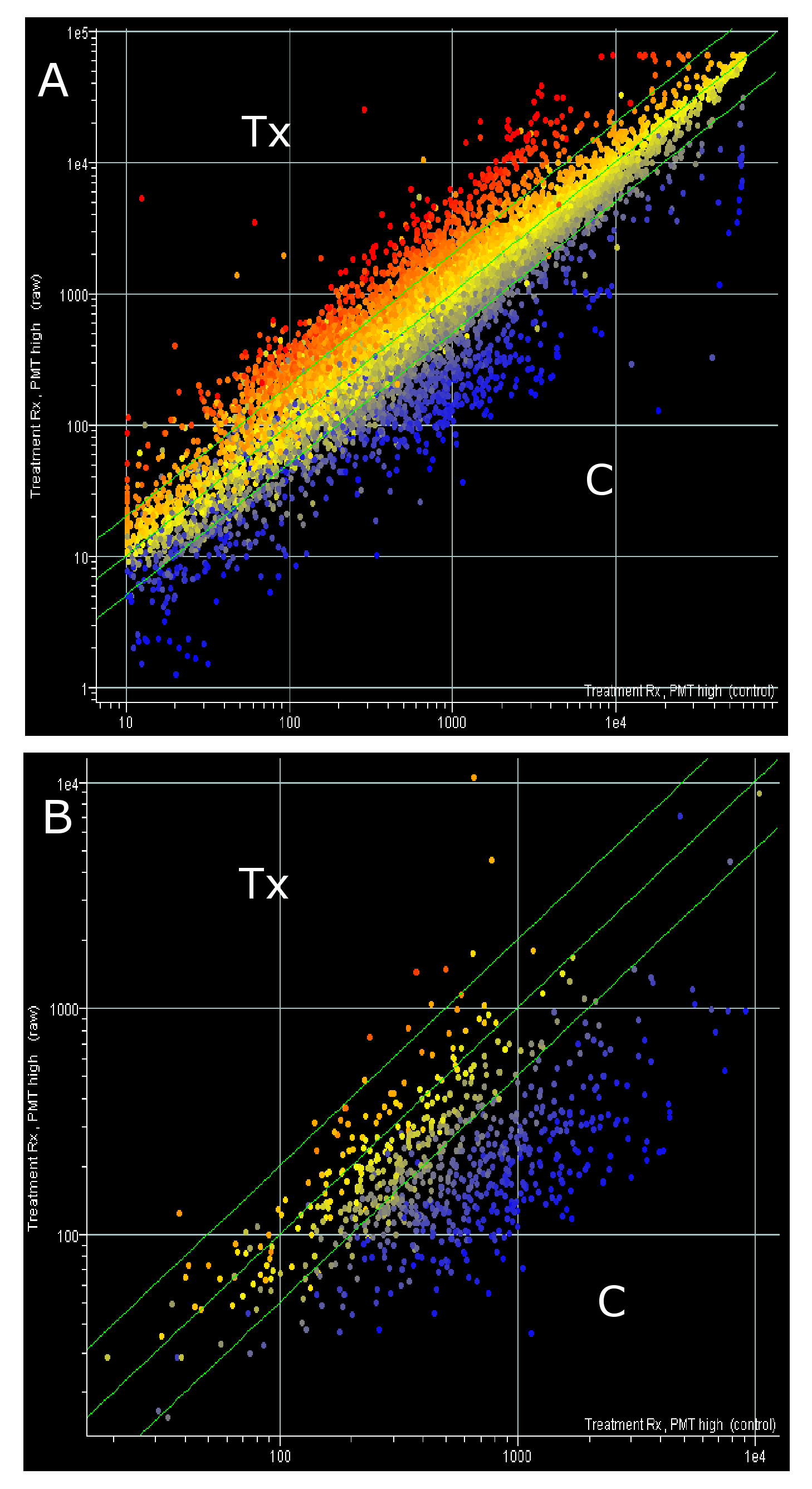

Supplement: Additional file 5 — Expression profiles for filarial and Wolbachia genes using the V2 filarial array after doxycycline treatment. Expression signals were documented by plotting the photomultiplier (PMT) signals at high intensities. Panel A (for all elements on the array) and panel B (Wolbachia elements only) are scatter plots displaying the mean normalized fluorescence intensity signals for elements in treated vs. control female worms. Up-regulated and down-regulated genes are shown in red and blue, respectively. 200 and 546 genes showing ≥ 2 fold change with P ≤ 0.01 were considered to be differentially expressed in Wolbachia and Brugia malayi after doxy treatment. Tx: treated; C: control. [file 1423-0127-19-21-S5.TIFF]

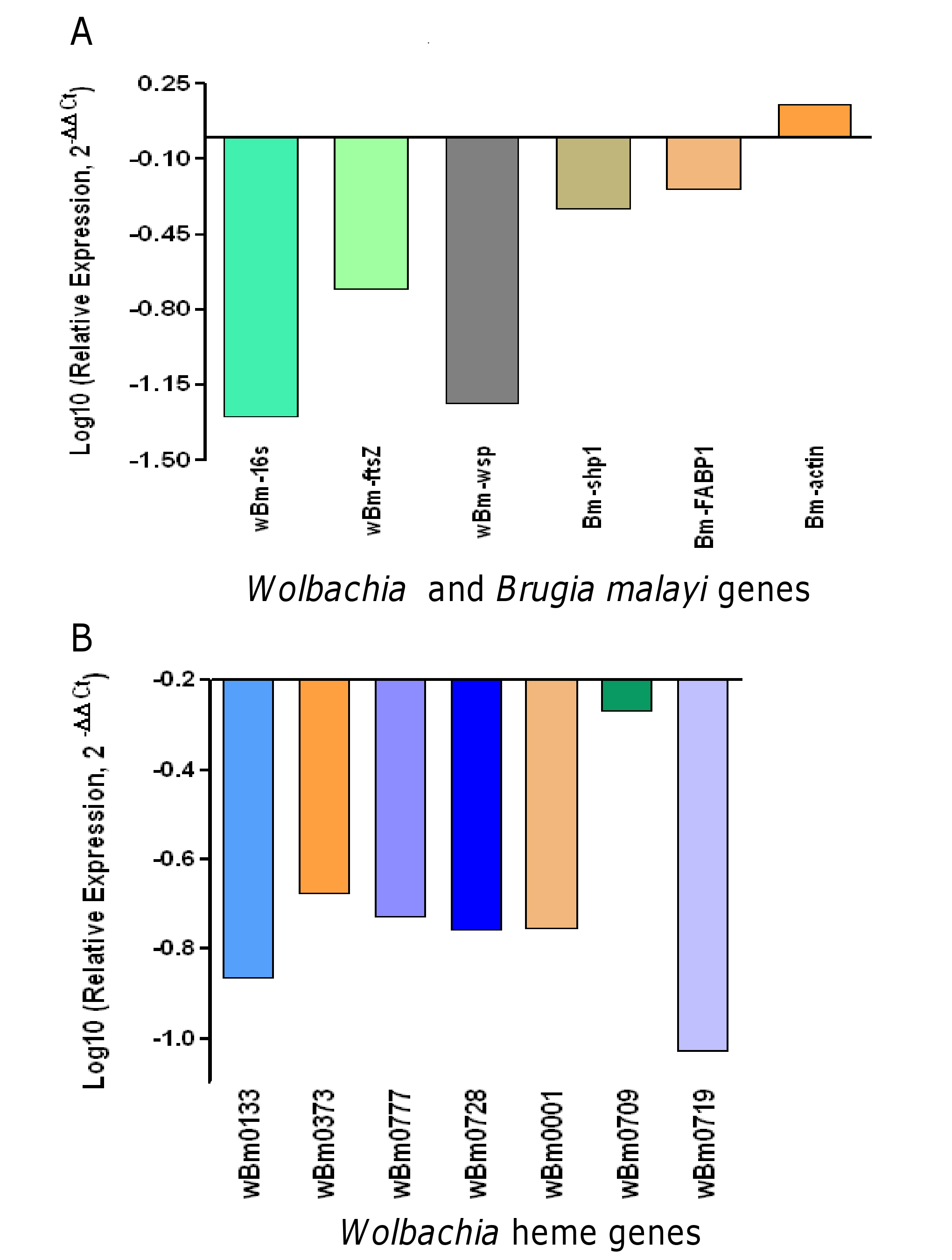

Supplement: Additional file 14 — Results of qRT-PCR assays for Wolbachia and Brugia malayi genes in female worms after doxycycline treatment relative to control worms. Panel A shows expression changes for wBm 16S rRNA, ftsZ and wsp transcripts and Bm-shp1, Bm-FABP1 and Bm-actin transcripts. Panel B shows changes in Wolbachia genes in the heme synthesis pathway. Expression profiles of 16S rRNA, ftsZ and wsp and Bm-shp1, FABP and heme genes relative to controls were significantly down-regulated (P < 0.05). [file 1423-0127-19-21-S14.TIFF]
