# Supplementary material for: Effects of Doxycycline on gene expression in Wolbachia and Brugia malayi adult female worms in vivo
Source: J Biomed Sci. 2012 Feb 9;19(1):21. doi: 10.1186/1423-0127-19-21 (PMC3352068; doi:10.1186/1423-0127-19-21)
Supplement: Additional file 2 — Distribution of Wolbachia and Brugia malayi elements in V2 filarial array hybridized with cDNA from doxycycline* treated or control worms. [file 1423-0127-19-21-S2.DOCX]

**Additional file** **2**. Distribution of *Wolbachia* and *Brugia malayi* elements in V2 filarial array hybridized with RNA from doxycycline*

treated or control female worms.

|  | *Wolbachia* | | *B. malayi* | |
| --- | --- | --- | --- | --- |
| Total number of elements | 804 | | 15412 | |
| Total number of elements detected in control  sample§ | 679 (84%) | | 13399 (87%) | |
| Total number of elements detected in  doxycycline treated sample^§^ | 538 (67%) | | 14340 (93%) | |
| Differentially expressed‍‍ genes^¥^ after  doxycycline treatment | 200 (37%) | Up: 3 (1.5%) | 546 (4%) | Up: 462 (84%) |
|  |  | Down: 197 (98.5%) |  | Down: 84 (16%) |

*Doxycycline was administered *p.o*. to *Brugia malayi* infected gerbils for 6 weeks at 100 mg/kg.

^§^ Total number of elements showing hybridization signals were from an average of 4 arrays and if any signal ≥ 250 or

signal/background ratio is ≥ 2 was considered as present.

^¥^ Genes showing normalized ratio (treated/control) of ≥ 2 fold change with a *P* value ≤ 0.01 were considered differentially

expressed.
